# Interrogating the Venom of the Viperid Snake *Sistrurus catenatus edwardsii* by a Combined Approach of Electrospray and MALDI Mass Spectrometry

**DOI:** 10.1371/journal.pone.0092091

**Published:** 2015-05-08

**Authors:** Alex Chapeaurouge, Md Abu Reza, Stephen P. Mackessy, Paulo C. Carvalho, Richard H. Valente, André Teixeira-Ferreira, Jonas Perales, Qingsong Lin, R. Manjunatha Kini

**Affiliations:** 1 Laboratório de Toxinologia, Instituto Oswaldo Cruz, Fiocruz, Rio de Janeiro, RJ, 21045–900, Brazil; 2 Department of Biological Sciences, 14 Science Drive 4, National University of Singapore, Singapore, 117543, Singapore; 3 School of Biological Sciences, University of Northern Colorado, 501 20th St., CB 92, Greeley, Colorado, 80639–0017, United States of America; 4 Laboratory for Proteomics and Protein Engineering, Carlos Chagas Institute, Fiocruz, Curitiba, PR, 81350–010, Brazil; 5 Department of Biochemistry and Molecular Biology, Medical College of Virginia, Virginia Commonwealth University, Richmond, Virginia, 23298–0614, United States of America; National Institutes of Health, UNITED STATES

## Abstract

The complete sequence characterization of snake venom proteins by mass spectrometry is rather challenging due to the presence of multiple isoforms from different protein families. In the present study, we investigated the tryptic digest of the venom of the viperid snake *Sistrurus catenatus edwardsii* by a combined approach of liquid chromatography coupled to either electrospray (online) or MALDI (offline) mass spectrometry. These different ionization techniques proved to be complementary allowing the identification a great variety of isoforms of diverse snake venom protein families, as evidenced by the detection of the corresponding unique peptides. For example, ten out of eleven predicted isoforms of serine proteinases of the venom of *S*. *c*. *edwardsii* were distinguished using this approach. Moreover, snake venom protein families not encountered in a previous transcriptome study of the venom gland of this snake were identified. In essence, our results support the notion that complementary ionization techniques of mass spectrometry allow for the detection of even subtle sequence differences of snake venom proteins, which is fundamental for future structure-function relationship and possible drug design studies.

## Introduction

Snake venoms not only represent rich sources of biologically active peptides and proteins but also serve as versatile platforms for the discovery and development of drug lead substances [1]. Significant progress in the investigations of snake venoms has recently been witnessed by different proteomics studies in this field. The combined transcriptome and proteome analysis of the venom of *Cerberus rynchops*, for example, revealed a very low complexity venom composition and a novel snake venom protein family called veficolins; function of veficolins has been hypothesized to be related to the inhibition of platelet aggregation [2]. Likewise, investigations into the venom of the ocellated carpet viper *Echis ocellatus* pointed to a pronounced role of transcriptional and posttranslational mechanisms on determining the final venom composition, as evidenced by a significant divergence between predicted toxin clusters found in the transcriptome and peptide sequences identified in the corresponding venom proteome [3]. A comparative proteome analysis of the venoms of terrestrial *Toxicocalamus longissimus* and a closely related marine species *Hydrophis cyanocinctus* indicates a pronounced reduction of the molecular diversity of the venom components of the marine snake as compared to the venom proteome of its terrestrial relative [4]. The authors reason that molecular economy of the toxin arsenal has been implemented as an evolutionary response to selective pressures from different environmental challenges. To predict possible structure function relationships of the various proteins of the corresponding venom, a complete picture of the sequences of the different protein families and their isoforms is of major importance. Extensive sequence coverage of the venom proteome can be accomplished using a combined approach of electrospray and MALDI ionization mass spectrometry. In the present study, we have used this approach to characterize the venom proteome of the pitviper *Sistrurus catenatus edwardsii* (Desert Massasauga Rattlesnake), a subspecies of *Sistrurus catenatus*, which is primarily encountered in dry and desert grasslands of the southwestern North American prairies [5, 6]. A comparative study of the venom proteomes of four different *Sistrurus* taxa has revealed an overview of the different protein families of the corresponding venoms, as evidenced by BLAST analysis of the detected sequences [7]. The transcriptome of the venom gland of *S*. *c*. *edwardsii* has also been characterized and serves as an exhaustive source for protein sequence investigations of the venom proteome [8]. Based on the identification of unique peptides of the corresponding proteins we were able to distinguish ten out of eleven predicted isoforms of serine proteinases and all five predicted metalloproteinase isoforms, together with a disintegrin. We also encountered the snake venom protein families C-type lectin, cysteine rich secretory protein, nerve growth factor, phospholipase A_2_, bradykinin-potentiating protein, and L-amino acid oxidase, previously described in the transcriptome of *S*. *c*. *edwardsii*. In addition, our analysis revealed the presence of snake venom protein families not detected in the venom gland transcriptome or previous studies, including glutaminyl cyclase, renin-like aspartic protease, and ecto-5'-nucleotidase. These results support the view that an in-depth analysis of the venom proteome is complementary to transcriptomic venom gland studies and will improve our understanding of the interplay of the different venom proteins on the target prey.

## Materials and Methods

### Venom extraction and Ethics statement

Specimens of *Sistrurus catenatus edwardsii* (Desert Massasauga) were collected in Lincoln County, Colorado, USA under permits granted by the Colorado Division of Wildlife to Stephen P. Mackessy (permits #0456, 06HP456). Venom was extracted manually [9] from 4 adult snakes from the same metapopulation in southeastern Colorado; venoms were pooled, centrifuged and lyophilized. Snakes were then PIT-tagged, returned to the exact locality and released. All procedures were permitted by the University of Northern Colorado Institutional Animal Care and Use Committee as detailed in UNC IACUC protocol #0702. No animals were sacrificed and no suffering of animals occurred during this study.

### Tryptic digestion of the venom

Lyophilized crude venom (600 μg) was initially dissolved in 600 μl of ammonium bicarbonate (50 mM) and precipitated with three volumes of ice-cold acetone for 3 h at -20°C. In the following, the sample was spun down at 14000 rpm for 10 min and the pellet brought up in 560 μl of ammonium bicarbonate (50 mM). Afterwards, 12 μl of 1% ProteaseMAX (in 50 mM ammonium bicarbonate) surfactant was added to the sample solution followed by reduction with 8 μl of 0.5 M DTT (at 56°C for 20 min) and alkylation with 16 μl of 0.55 M iodoacetamide at room temperature for 30 min in the dark. Finally, the venom was subjected to digestion (at 37°C for 12 h) by adding 10 μl of trypsin (1 μg/μl in 50 mM acetic acid) and 6 μl of 1% ProteaseMAX surfactant to enhance the enzymatic performance of trypsin.

### Chromatography and mass spectrometry

The tryptic digest was separated on a C-18 reversed phase column (Agilent Zorbax 300SB-C18 1.0 x 150 mm x 3.5 μm) by running a linear gradient from 0% acetonitrile to 72% acetonitrile in 120 min applying a flow rate of 40 μl/min (solvent A contains H_2_O / 0.1% TFA, solvent B contains 80% ACN / 0.1% TFA) on a LC Packings Ultimate HPLC system (Dionex, Vernon Hills, IL). During the chromatographic run approximately 130 fractions were manually collected in Eppendorf vials. After reducing the volume in each vial to approximately 1 μl on a Speedvac (Savant SC 110A), samples were spotted onto the MALDI sample plate. Approximately 0.3 μl of the sample solution was mixed with the same volume of a saturated matrix solution (α-cyano-4-hydroxycinnamic acid, (Aldrich, Milwaukee, WI) 10 mg/ml in 50% acetonitrile/0.1% trifluoroacetic acid) on the target plate and allowed to dry at room temperature (dried-droplet method). Raw data for protein identification were obtained on a AB Sciex 5800 (AB Sciex, Foster City, CA) and a 4700 Proteomics Analyzer (Applied Biosystems, Foster City, CA). Typically, 1600 shots were accumulated for spectra in MS mode while 3500 shots were accumulated for spectra in MS/MS mode. Up to twenty of the most intense ion signals with a signal to noise ratio above 2 were selected as precursors for MS/MS acquisition excluding common trypsin autolysis and keratin peaks. External calibration in MS mode was performed using a mixture of six singly charged peptides: des-Arg1-Bradykinin (m/z = 904.468), angiotensin I (m/z = 1296.685), Glu1-fibrinopeptide B (m/z = 1570.677), ACTH (1–17 clip) (m/z = 2093.087), ACTH (18–39 clip) (m/z = 2465.199), and ACTH (7–38 clip) (m/z = 3,657.929). MS/MS spectra were externally calibrated using known fragment ion masses observed in the tandem mass spectrum of Glu1-fibrinopeptide B.

Tryptic peptides were also separated on an Easy nLC II (Thermo Scientific) nanoflow HPLC system connected to an LTQ-Orbitrap XL mass spectrometer (Thermo, Bremen, Germany) equipped with a nanoelectrospray ion source. Peptides were initially loaded onto a trap column (100 μm x 2 cm) packed in-house with C_18_ resin (5 μm, 100 Å pore, Magic C_18_ AQ, Bruker-Michrom, Auburn, CA) and separated on an RP HPLC column (C_18_, 75 μm x 30 cm) using a linear gradient from 98% solvent A (H_2_O, 0.1% formic acid) to 60% solvent B (ACN, 0.1% formic acid) over 162 min. Precursor scans were performed in the Orbitrap mass detector at a resolution of 60,000 in the mass range of 300 m/z to 1700 m/z, while MS/MS scans were acquired in the linear trap (“high-low”). With an exclusion of singly charged ions, up to ten of the most intense precursor ions were subjected to product ion scans using CID with a normalized collision energy of 35%. Moreover, MS/MS scans were only triggered for precursor ions having a minimum signal threshold of 10,000 counts. Precursors that were selected for MS/MS scans were dynamically excluded for 30 sec from a repeated product ion scan within a ±10 ppm mass error. Different HPLC separations were performed where the fragmentation of precursor ions was induced using CID only as well as an approach of alternating CID and ETD, in which successively the same precursor ion was fragmented by CID and ETD.

### Data analysis

Database searches of the mass spectra acquired on the MALDI mass spectrometers were searched against all entries of NCBInr (www.ncbi.nlm.nih.gov/index.html) using the Mascot software (www.matrixscience.com) and against an in-house created snake venom database using Mascot (Mascot, version 2.1). The following search parameters were used: No restrictions on species of origin or protein molecular weight, semi-tryptic cleavage products, two tryptic missed cleavages allowed, variable modifications of cysteine (carbamidomethylation) and methionine (oxidation), and pyroglutamate formation at N-terminal glutamine of peptides.

Electrospray data were analyzed using the Peaks (Peaks Studio 5.3) and ProLuCID [10] search engines, respectively, against an in-house created snake venom database (2580 entries). The search parameters of the Peaks search engine were sulfation (serine, threonine), phosphorylation (serine, threonine, and tyrosine), deamidation (glutamine, asparagine), dehydration (serine, threonine) oxidation (methionine, tryptophan, and histidine) and acetylation at N-terminal of peptides, with a maximum number of 3 modifications per peptide allowed. Search parameters of ProLuCID were fixed modification of cysteine (carbamidomethylation), variable modifications of methionine (oxidation), a precursor tolerance of 50 ppm and allowance for semi-tryptic identifications. Peptide spectrum matches obtained by ProLuCID were then validated by the Search Engine Processor with the default parameters previously described [11]. All identified peptides were further manually verified.

## Results and Discussion

**Fig 1:**
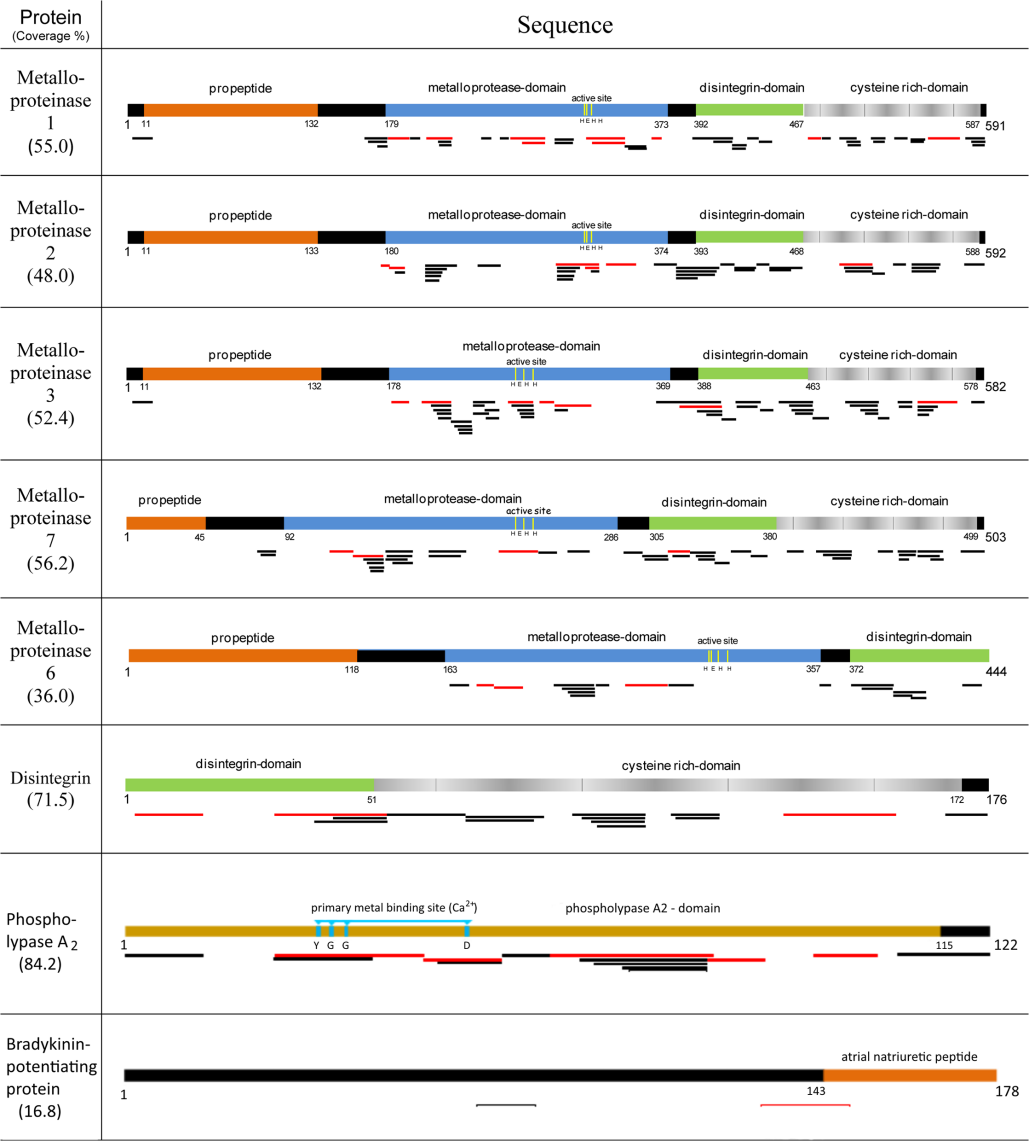
Sequence coverages of some of the predicted venom gland proteins of *S*. *c*. *edwardsii* as revealed by the combined approach of MALDI and ESI tandem mass spectrometry. Protein sequences including specific domains are indicted by colored bars; below these, corresponding peptides identified by ESI (black lines) and MALDI (red lines) are indicated.

**Fig 2:**
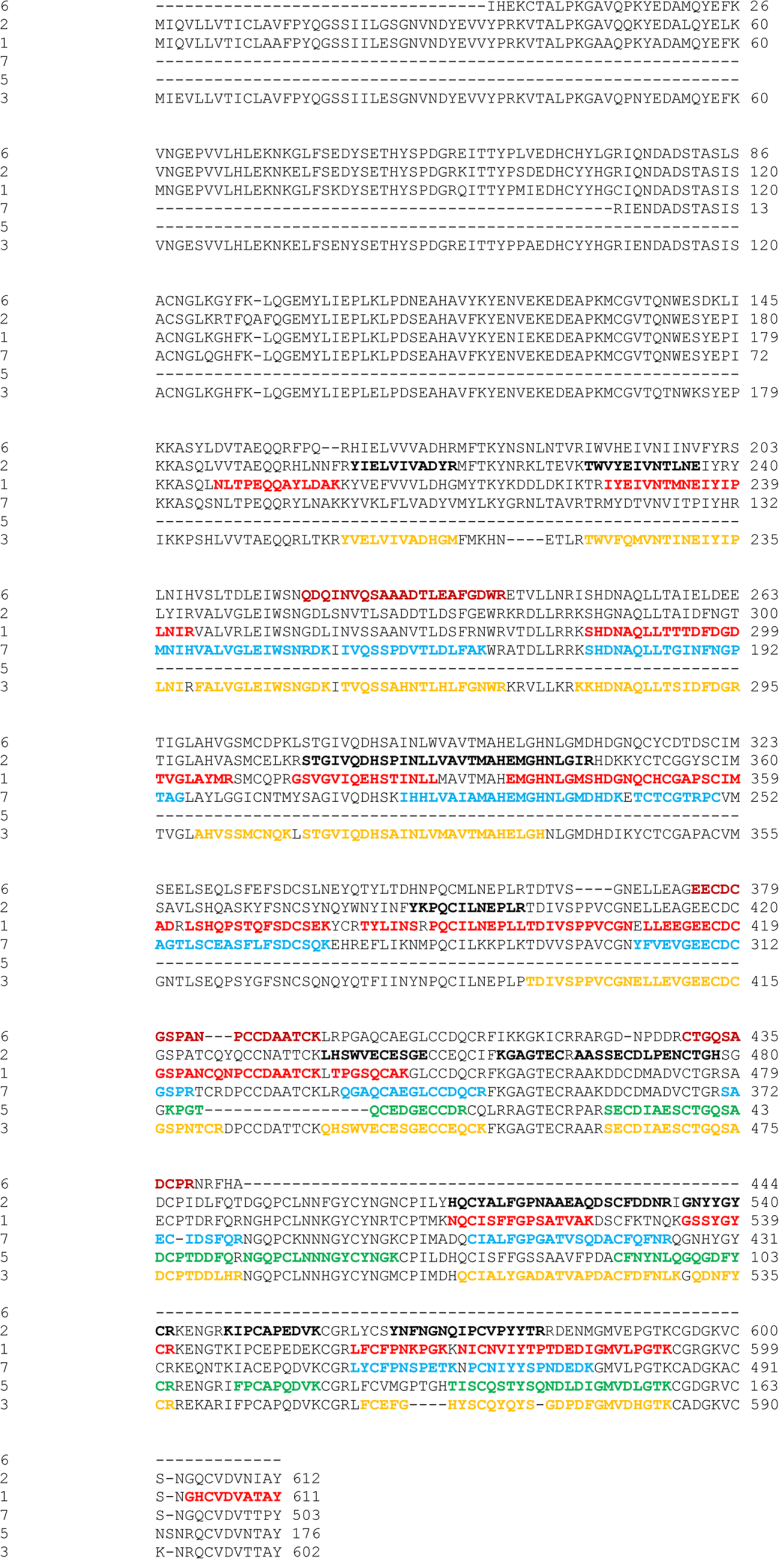
Sequence alignment of the metalloproteinases of the venom of *S*. *c*. *edwardsii*. Different colors indicate the unique peptides identified by tandem mass spectrometry of the corresponding proteins.

Envenomation by viperid snakes frequently manifests as a complex medical syndrome dominated by hemorrhagic and inflammatory processes triggered by the combined enzymatic actions of metalloproteinases, serine proteinases and phospholipases A_2_ [12–14], as well as by the detrimental effects of C-type lectins (CLP) on platelet function [15]. The snake venom metalloproteinases (SVMP) are classified in four different major groups (PI, PII, PIII, and PIV) based on their final domain composition after posttranslational modification of the corresponding multidomain precursor protein [16, 17]. Functionally, a broad spectrum of biological activities have been attributed to SVMPs, including hemorrhagic, inflammatory and myonecrotic effects [14, 18]. To date, only a few studies have noted the presence of peptides related to the prodomain of SVMPs in the venom [19, 20] and it might be that in most cases the prodomain of the precursor protein is enzymatically removed before its secretion into the venom gland. Only a single (identical) peptide of the prodomain of SVMP isoforms 1 and 3 (Fig 1) was identified, suggesting that the prodomain is proteolytically removed in *S c*. *edwardsii* before being exocytosed to the venom gland lumen. The metallo-, disintegrin-, and cysteine rich domains of the four isoforms that belong to the PIII class of metalloproteinases revealed evenly distributed sequence coverage (Fig 1), supporting the view that these domains are efficiently translated. Similarly, we were able to identify tryptic fragments of the predicted [8] PII (isoform 6) and disintegrin of the venom proteome, with sequence coverages of 36% and 71%, respectively (Fig 1). The presence of proteotypic peptides of the corresponding isoforms clearly revealed the existence of these different proteins in the venom proteome (Fig 2). Of further particular note is the presence of protein sequences that went undetected in the transcriptome analysis but could be identified in the venom proteome. These sequences belong to 26 different SVMPs identified as PII and PIIIs and disintegrins of snakes phylogenetically closely related to *S*. *c*. *edwardsii* (Table 1). Interestingly, during a recent investigation of the transcriptome and proteome of the cryptic snake *Drysdalia coronoides*, the SVMPs of the venom were initially only identified in the proteome and only the implementation of gene-specific 3’RACE primers of the corresponding signal peptides of the targeted proteins revealed the cDNA sequence [21]. These findings might point to a general difficulty to characterize the relatively large SVMP sequences fully in the transcriptome of the venom gland without the use of amplification techniques. However, it is worth mentioning that a recent in-depth transcriptome analysis of the venom of the eastern diamond rattlesnake *Crotalus adamanteus* produced full-lengths SVMP sequences by using next-generation sequencing including the Illumina technology [22] The high abundance of metalloproteinases in the venom of *S*. *c*. *edwardsii* is in line with a previous transcriptome analysis [8] and point to an explicit role of accelerated evolution on the development of distinct metalloproteinase isoforms [23].

**Table 1:** Identification of proteins of the venom of *S*. *c*. *edwardsii* not predicted from the transcriptome analysis.

Protein family	Taxonomy	gi	Sequence	charge	m/z	score
PI-Metalloproteinase	Agkistrodon contortrix laticinctus	1098019	QWVHQIVNTINEIYR	2	958	34.93
PII-Metalloproteinase	Bothrops jararaca	123911605	LTTGSQCAEGLCCDQCK	1	1987.75	**130**
			DSCSCGANSCIMSATVSNEPSSR	1	2476.95	**154**
PII-Metalloproteinase	Echis ocelatus	320579329	YVQLVIVADHSMVTK	1	1703.83	24.89
PII-Metalloproteinase	Crotalus adamanteus	338855314	YHFVANR	1	906.46	**53**
			NSVGIVQDHR	1	1124.59	**48**
PII-Metalloproteinase	Glodius saxatilis	31322301	YNSNLDTIR	2	548.28	54.37
PII-Metalloproteinase	Crotalos atrox	258618062	VALIGLEIWSSGELSK	1	1702.97	*0*.*65*
			YYTEVGEDCDCGPPANCQNPCCDAATCK	1	3312.36	**80**
PIII-metalloproteinase	Botrox atrox	205278807	SVGIVQDHGK	2	347.19	65.47
			HELGHNLGMDHDR	1	1546.71	**94**
			SYQFSDCSQNDHLR	1	1757.76	*0*.*53*
			YLISHTPQCILNEPLR	2	977.53	75.23
PIII-metalloproteinase	Trimeresurus gramineus	172044536	AQCGEGLCCDQCR	2	1613.61	*0*.*32*
			SCAGQSADCPTDDFHR	2	912.37	73.56
			AGEDCDCGSPANPCCDAATCK	1	2258.73	32.37
PIII-Metalloprotease	Glodius halys	4106007	AAGDTLEAFGDWR	2	704.84	63.91
			LRPGQQCAEGLCCDQCR	1	2107.9	*0*.*54*
PIII-Metalloprotease	Agkistrodon psicivorus leucostoma	258618068	YLIDNRPPCILNK	2	808.45	41.89
			NLQGQGNFYCR	1	1356.66	**46**
PIII-Metalloproteinase	Atractaspis microlepidota andersoni	6007789	DTLDSFEEWR	2	649.3	52.74
PIII-metalloprotease	Bothrops jararaca	209870468	HDNAQLLTAIDFNGR	2	843.43	49.11
PIII-metalloprotease	Viridovipera stejnegeri	123900232	QLLTAIDFDGPTIGR	2	808.95	66.65
			LHSWVECESGECCEQCR	1	2226.88	*0*.*61*
PIII-Metalloproteinase	Trimeresurus flavoviridis	82217336	QGNYYGYCR	1	1222.5	30.19
PIII-Metalloproteinase	Bothrops jararacussu	123889624	YSEDLDFGMVDHGTK	1	1729.7	36.49
PIII-Metalloproteinase	Echis coloratus	297593938	VTLNSFGEWR		1208.58	30.30
PIII-Metalloproteinase	Echis carinatus sochureki	297593788	LHSWVECESGECCDQCK		2183.82	38.67
PIII-Metalloproteinase	Bothrops jararaca	82219706	SECDIAESCTGQSADCPTDDFKR	1	2649.01	**214**
PIII-Metalloproteinase	Crotalus atrox	75570463	SECDIAESCTGQSADCPTDDFHR	1	2658.05	**185**
PIII-Metalloproteinase	Crotalus adamanteus	338855314	YEGDKTEICSR	1	1357.58	**32**
			MAHELGHNLGIDHDR	1	1714.76	**51**
PIII-Metalloproteinase	Trimeresurus flavoviridis	344925813	HSVGIVQDHGK	1	1176.59	**44**
PIII-Metalloproteinase	Crotalus adamanteus	338855316	LDVMVAVTMAHELAH	1	1636.80	**125**
PIII-Metalloproteinase	Crotalus adamanteus	338855326	YSEDLDYGMVDHGTK	1	1729.73	**114**
			LFCKFNNFPCQYK	1	1766.79	**72**
			LHSWVECESGECCEQCK	1	2197.83	**108**
PIII-Metalloproteinase	Crotalus adamanteus	338855330	PKCILNEPLR	1	1239.65	**64**
			TDIISPPVCGNELLEAGEECDCGSPR	1	2875.28	**120**
disintegrin	Gloydius shedaoensis	91680863	CTGQSAECPTDDFHR	2	890.86	57.51
			YFVEVGEECDCGLPAHC	1	2041.84	*0*.*54*
			SECDIAESCTGQSAECPTDDFHR	1	2672.07	**185**
disintegrin	Crotalus atrox	327507705	GDWNDDTCTGQSADCPR	1	1954.75	**131**
Serine proteinase	Crotalus adamanteus	338855332	AAYPEFGLPATSR	2	690.36	66.03
Serine proteinase	Bothrops jararaca	82233395	LDSPVSDSEHIAPL	2	740.38	42.25
Serine proteinase	Viridovipera stejnegeri	82242793	IIGGDECNIDEHR	2	764.36	56.68
C-type lectin	Sistrurus miliarius	21530567	GLQQGTNYHK	2	382.53	72.72
			FCSEQAEGGHLVSIESSEEAA	1	2239	*0*.*54*
			WSDGSSVSYENWIEAESK	1	2073.83	**125**
			DCPSGWSSYDQHCYR	1	1917.74	**118**
C-type lectin	Sistrurus miliarius	21530570	YDVWIGLR	2	511.28	60.77
			WSDGSSVNYENLIK	2	806.4	75.31
			DFDCPSDWYAYDQYCYR	1	2323.83	**144**
C-type lectin	Sistrurus miliarius	21530564	FTSMWIGLK	1	1082.57	**64**
			LASIHSSEEEAFVGK	1	1603.81	*0*.*52*
			TWDDAESFCYTQHR	1	1815.69	**95**
C-type lectin	Sistrurus miliarius	21530573	QNQYYVWIGLR	1	1439.75	**53**
			ETEFLQWYNTDCEEK	1	1991.88	**84**
C-type lectin	Crotalus adamanteus	338855278	YEDWAEESYCVYFK	1	1888.79	**92**
C-type lectin	Crotalus durissus terrificus	82129809	WSDGSSVNYENLLK	2	806.40	75.31
			QNKYYVWIGLR	1	1439.75	**46**
			ETEFLQWYNTDCEEK	1	1991.86	**75**
L-amino acid oxidase	Bothrops neuwiedi pauloensis	195927838	GNPLEECFR	2	561.26	59.52
			NGLSATSNPK	2	495.25	45.52
L-amino acid oxidase	Demansia vestigiata	118151720	YPVKPSEK	2	474.27	42.82
L-amino acid oxidase	Viridovipera stejnegeri	34014953	LSAAYVLAGAGHEVTVLEASER	1	2244.2	*0*.*56*
L-amino-acid oxidase	Naja kaouthia	124015192	QNDYEEFLEIAK	2	749.86	59.53
L-amino-acid oxidase	Crotalus atrox	124106294	TPYQFQHFSEALTAPFK	1	2012.03	**80**
CRISP	Crotalus atrox	28972959	EDKYTNCK	1	1057.43	**53**
			SLVQQAGCQDK	2	617.31	75.45
			MEWYPEAAANAER	1	1537.68	**105**
			SGPPCGDCPSACDNGLCTNPCTK	1	2525.08	**152**
CRISP	Vipera nikolskii	215262114	GNVDFDSESPR	1	1263.55	**74**
Venom nerve growth factor	Bothrops asper	186659795	NPNPVPTGCR	2	556.28	52.27
Venom nerve growth factor	Cryptophis nigrescens	123907150	HWNSYCTTTQTFVK	1	1773.81	**96**
Phospholipase A2	Sistrurus catenatus tergeminus	45934756	LDTYTYSEENGEIICGGDDPCKK	1	2664.18	**124**

**Fig 3:**
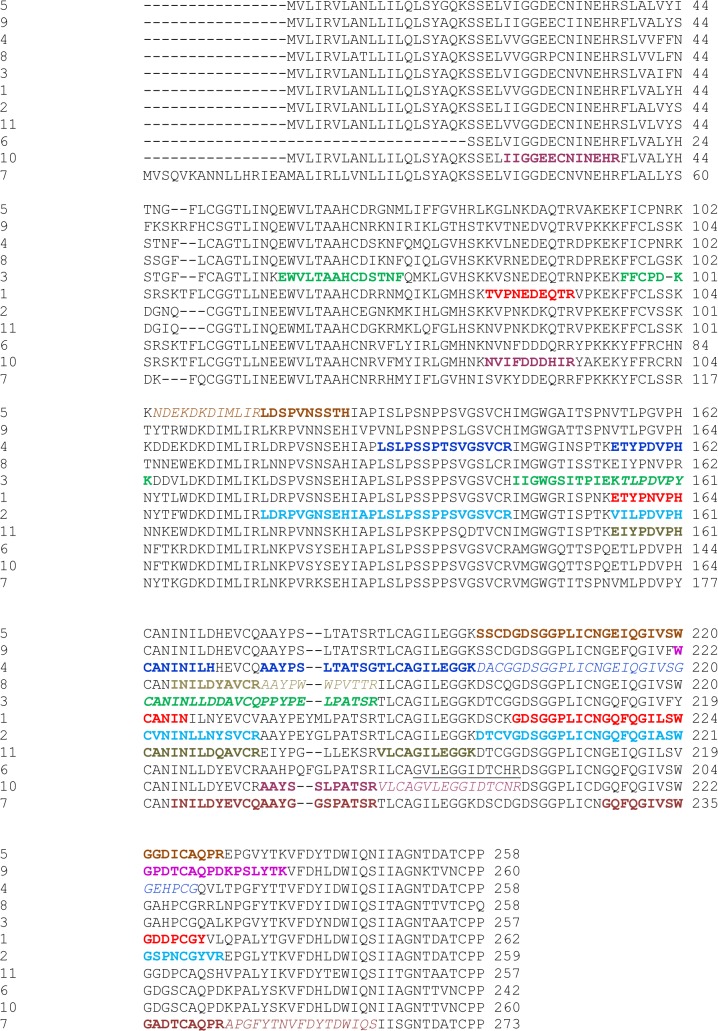
Sequence alignment of the serine proteinases of the venom of *S*. *c*. *edwardsii*. Different colors indicate the unique peptides identified by tandem mass spectrometry of the corresponding proteins.

Snake venom serine proteinases (SVSPs) primarily affect the hemostatic system of prey organisms and often show fibrinogenolytic and fibrinolytic activities. Since this is similar to the action of thrombin on fibrinogen, SVSPs have also been known as thrombin-like enzymes (TLEs) [24, 25]. In addition, SVSPs also act on kininogen and platelet receptors [26]. While most SVSPs exist as monomers, dimeric forms have been detected in the venom of the viper *A*. *b*. *brevicaudus* [27]. One of these is brevinase, a heterodimeric enzyme with a covalent disulfide link between the two monomers. The analysis of the transcriptome of the venom gland of *S*. *c*. *edwardsii* predicted the presence of eleven SVSP-specific isoforms. Previous studies on the protein coding region of SVSPs in pitvipers have noted a trend towards accelerated evolution of this protein family, a result which is also found in the venom of *S*. *c*. *edwardsii*, as evidenced by the ratio (0.99) between non-synonymous and synonymous substitutions of the exon sequences of the mRNA transcripts [8]. Such sequence variations are likely related to different pharmacological activities. The combined electrospray and MALDI ionization analysis of the proteome resulted in the identification of ten out of eleven predicted SVSP isoforms, as evidenced by the detection of unique peptides (Fig 3) with a sequence coverage between 35% (isoform 8) and 82% (isoform 1). Again, we observed the presence of additional peptides that match sequences of SVSPs from related snake venoms (*e*. *g*. *C*. *adamanteus*) and that were not identified in the transcriptome analysis of *S*. *c*. *edwardsii* (Table 1). Taken together, these additional ten identified isoforms raises the total number of isoforms of SVSPs to a total number to twenty, reinforcing the idea that this protein family has evolved in an accelerated manner, producing an elevated number of isoforms.

Phospholipase A_2_ (PLA_2_s) are functionally characterized by their multiple pharmacological activities such as cardiotoxic, neurotoxic, myotoxic, antiplatelet and anticoagulant effects [28, 29]. They enzymatically cleave the second ester bond of the glycerol ester and represent one of the most extensively studied snake venom families. The venom proteome revealed the presence of only one PLA_2_ protein (Fig 1), consistent with the transcriptome analysis of the venom gland [8]. However, an additional single peptide that showed sequence identity to a PLA_2_ from *S*. *c*. *tergeminus* (Table 1) was also found. It appears that, contrary to other species, where PLA_2_s are present in multiple isoforms, the venom of *S*. *c*. *edwardsii* was not under evolutionary pressures selecting for the evolution of a pronounced diversity of PLA_2_s.

The cysteine-rich-secretory proteins (CRISP) are widely distributed in snake venoms, particularly in Viperidae and Elapidae. The biological activity of some is related to the inhibition of the cyclic nucleotide-gated ion channels as well as L-type Ca^2+^ and BK_Ca_ K^+^ channels [30]. For example, triflin and ablomin (from the pitviper *Gloydius blomhoffii*) block L-type Ca^2+^ channels that lead to contraction of smooth muscle [31, 32]. However, scientists are only beginning to understand the full scope of biological and pharmacological effects of this protein family. We identified the predicted CRISP protein in the venom of *S*. *c*. *edwardsii* (60% coverage) along with peptides that match part of the CRISP protein Catrin (gi 28972959) from *C*. *atrox* [33]. Both proteins share about 87% sequence identity, with pronounced variations located primarily at the C-terminus. In addition, a peptide that matches a CRISP protein from another viperid species was also encountered.

The snake C-type lectin or C-type lectin-like protein families (snaclecs [34]) usually form disulfide linked homo- or hetero-dimers which are organized in oligomers to form larger quaternary protein complexes [35]. They affect the haemostatic system by interfering with coagulation factors or platelet activation [15]. The analysis of the proteome of *S*. *c*. *edwardsii* led to the identification of three isoforms of C-type lectins, as evidenced by the detection of sequence-specific unique peptides with sequence coverages between 67% (isoform 1) and 24% (isoform 3). Interestingly, however, the presence of peptide sequences identical to those of six C-type lectin proteins from the closely related rattlesnakes *Sistrurus miliarius*, *Crotalus adamanteus*, and *Crotalus terrificus* were also noted (Table 1). This brings the number of C-type lectins in the venom of *S*. *c*. *edwardsii* to a total of nine isoforms and might indicate a prominent role of this protein family to the envenomation of prey by *S*. *c*. *edwardsii*.

Snake venom L-amino acid oxidases (SV-LAAOs) catalyze the oxidative deamidation of amino acids and, besides effects on platelet aggregation, may induce apoptosis in prey [36]. We identified the predicted SV-LAAO (65% coverage) from *S*. *c*. *edwardsii* and also found five additional SV-LAAOs with sequence identities related to viperid and elapid snakes; an investigation of the biological functions of these isoforms in the venom could illuminate a broader role for SV-LAAOs in envenomation.

Bradykinin-potentiating peptides (BPPs) inhibit the activity of angiotensin I-converting enzyme (ACE) by repressing both the generation of the hypertensive peptide angiotensin II as well as the degradation of the hypotensive peptide bradykinin [37]. The result of these synergistic actions is a significantly reduced blood pressure in envenomated animals [38]. The transcriptome investigation of *S*. *c*. *edwardsii* revealed only one singleton (transcript abundance 0.28%) encoding for BPPs. This low abundance is in line with the modest sequence coverage (16.8%, Fig 1) [8] obtained via proteome analysis and it appears that contrary to other pitvipers such as *Bothrops* and *Lachesis*, BPPs play a minor role in envenomation by *S c edwardsii*.

Vascular endothelial growth factors from snake venoms (VEGF-F) bind specifically the kinase insert domain-containing receptor (KDR) and thereby induce low blood pressure as well as proliferation of vascular endothelial cells [39, 40]. In the venom of *S*. *c*. *edwardsii* we identified the predicted VEGF-F together with two peptides that showed homology to VEGF-Fs from the viper *B*. *asper* (Central America) and the elapid *C*. *nigrescens* (eastern small-eyed snake; coastal eastern states of Australia). Again, there appears to be greater diversity in the proteome of *S*. *c*. *edwardsii* than was previously observed.

Several protein families were identified in the current study which were not found in the previous transcriptome analysis of the venom gland of *S*. *c*. *edwardsii*. The cyclization of N-terminal glutamine by glutaminyl cyclase (QC) is an important posttranslational process in the modification of a variety of proteins including hormones and cytokines [41], and this modification is found in many venom proteins, including SVMPs and some colubrid three-finger toxins [42]. The formation of pyroglutamine at the N-terminal likely protects proteins from enzymatic degradation and induces conformational changes to improve receptor binding [43]. Recently, glutaminyl cyclase was found in the venom gland of colubrid snakes (*Boiga*) and the authors propose that this modification might lead to increased stability of venom components against exopeptidase degradation and therefore indirectly contributing to venom toxicity [44]. The QC encountered in the venom of *S*. *c*. *edwardsii* might have similar functions (Table 2). Interestingly, recent proteomic studies of the venoms of rattlesnakes of the *Crotalus species*, which is related to *S*. *c*. *edwardsii*, also revealed the presence of glutaminyl cyclase [45–47].

**Table 2:** Identification of protein families of the venom of *S*. *c*. *edwardsii* not predicted from the transcriptome analysis.

Protein family	Taxonomy	gi	Sequence	charge	m/z	score
Ecto-5'-nucleotidase	Gloydius blomhoffi	211926756	SSGNPILLNK	2	521.80	73.51
			ETPVLSNPGPYLEFR	1	1718.83	**95**
			LTAVLPFGGTFDLLQIK	1	1834.08	*0*.*58*
Glutaminyl cyclase	Gloydius blomhoffi	15991080	LIFFDGEEAFVR	2	721.88	75.11
			TFSNIISTLNPLAK	2	759.94	69.35
			WSPSDSLYGSR	2	627.8	54.71
			FVLLDLIGAR	2	558.85	52.56
			NTYQIQGIDLFVLLDLIGAR	1	2263.29	*0*.*53*
Renin-like aspartic protease	Echis ocellatus	109287598	GFLSQDIVR	1	1034.57	0.49
Phospholipase B	Crotalus adamanteus	338855308	VVPESLFAWER	1	1332.72	**74**
			HGLEFSYEMAPR	1	1436.66	**97**
			NGYWPSYNIPFDK	1	1600.77	**67**
			HQGLPESYNFDFVTMKPVL	1	2222.07	**48**

Peptides scores of the different search engines are: Peaks—regular, Mascot—**bold**, and ProLuCID—*italics* letters.

We also found in the venom proteome three peptides that showed identity to ecto-5'-nucleotidase (5' NT) from *Gloydius blomhoffi* venom. Nucleotidases from different snake venoms have been functionally related to the inhibition of platelet aggregation [48, 49]. A study using mouse and human blood revealed that 5' NT from *Crotalus atrox* inhibits platelet aggregation via the production of increased levels of extracellular adenosine [50].

Renin-like aspartic protease was described for the first time in the venom gland transcriptome of *Echis ocellatus*, a viperid snake found in West Africa [51]. Based on the confident identification of a single peptide we confirm the presence of this enzyme in the proteome of the venom of *S*. *edwardsii*. To date, there are no further descriptions in the literature on the potential function of this protein, and it would be interesting to investigate the biological implications of this enzyme, especially on venom potency.

Phospholipase B (PLB) cleaves ester linkages from both the sn-1 and sn-2 positions of glycerophospholipids. Recently, a PLB has been identified for the first time in the venom of the cryptic snake *Drysdalia coronoides* [21]. While the three dimensional structure of this enzyme have not yet been resolved, it is known to form both monomers and dimers. The peptides of the PLB encountered in the venom of *S*. *c*. *edwardsii* show identity to the PLB detected in the transcriptome of the venom gland of the related rattlesnake *C*. *adamanteus*. The entire protein sequence of the PLB in the venom of *C*. *adamanteus* shows 553 amino acids, including a 27 residue signal peptide and a 526 amino acid phospholipase B domain [52]. Elucidation of the structure and function(s) of this protein from *S*. *c*. *edwardsii* venom may reveal diverse phospholipase subtypes in this venom and help explain the lack of PLA_2_ diversity. It is interesting to compare the sequences identified in the venom of *S*. *c*. *edwardsii* in the present study with the sequences detected by Edman degradation and *de novo* sequencing of MS/MS spectra in the same venom in the study by Sanz and coworkers. While we were able to match nearly all of the sequences determined by N-terminal sequencing (S3 Table) in the study by Sanz *et al*. [7] to protein snake venom families of *S*. *c*. *edwardsii* identified in the present work, peptide sequences inferred by *de novo* sequencing (S3 Table) were more difficult to match. Interestingly, many sequences in the paper by Sanz *et al*. refer to SVMP’s (S2 Table), which we were not able identify in the venom of *S*. *c*. *edwardsii*, in spite of the fact that both studies utilized venom from the same source population. This might point to venom heterogeneity among this population of snakes occurring in a rather limited area (~1600 hectares).

The relatively high abundance of metalloproteinases in the venom of *S*. *edwardsii*, which have the ability to cleave extracellular matrix and other structural proteins, indicate that the envenomation of prey is primarily related to hemorrhagic/tissue damaging events rather than myotoxic effects. This conclusion is also supported by the observation that specific small peptide myotoxins, such as myotoxin a from *C*. *viridis viridis* venom [53], and prominent PLA_2_ myotoxins [54], appear to be absent from the venom. Human envenomations by *Sistrurus catenatus* are uncommon; for example, only 9/650 reported snakebites resulted from *S*. *catenatus* [55]. Similarly, case reports are rare, but the clinical presentation is considered to be similar to *Crotalus* sp. bites, requiring antivenom treatment but typically with less severe outcome [56, 57]. Bites by *S*. *c*. *edwardsii* are even less frequent, but because this species has a toxic venom (mouse LD_50_ = 0.60 μg/g; [58]) which contains abundant serine proteases, coagulopathies including hypofibrinogenemia and thrombocytopenia are to be expected.

### Proteome of the venom of *S*. *c*. *edwardsii*

The sequence coverages accomplished by mass spectrometry of the different venom proteins range from 16.8% (BPP) to 82% (PLA_2_). This distribution is reflected in the corresponding transcriptome analysis of the venom of *S*. *c*. *edwardsii*, in which the BPP represents the lowest abundance protein (0.28%) and the PLA_2_ the highest abundance protein (28.06%) of the single protein identifications. The SVMPs of the venom of *S*. *c*. *edwardsii* reveal moderate sequence coverage of up 56% that might be related to the fact that the prodomain of the corresponding isoforms is included in the sequence of the mature proteins. However, the prodomain of SVMPs might be proteolytically cleaved *before* secretion into the venom gland lumen [16]. In this case, the sequence of the mature SVMPs would lack the prodomain, hence, the coverage would significantly increase. The differentiation of multiple isoforms of proteins by mass spectrometry is particularly demanding due to pronounced sequence homology, as is the case with SVSPs from the venom of *S*. *c*. *edwardsii*. However, we were able to distinguish ten out of eleven predicted isoforms of SVSPs (Fig 2) based on the identification of unique peptides of the corresponding proteins. The complementary use of ESI and MALDI ionization techniques leads to increased sequence coverage of the proteins investigated, compared to the sole application of one of these techniques [59, 60]. Indeed, inspection of the peptides detected revealed the identification of different sets of peptides of the corresponding proteins depending on the ionization technique applied. In some cases, such as the C-type lectin isoforms and the vascular endothelial growth factor, peptides were predominantly identified by MALDI, while other proteins like CRISP and the L-amino acid oxidase were detected by the identification of tryptic peptides ionized primarily by ESI. Different studies have shown that in MALDI experiments, peptides containing arginine residues generated through tryptic digestion are preferentially ionized compared to peptides that carry a lysine residue [61–63]. This has been related to the increased gas phase basicity of arginine compared to lysine. During the course of the manual MS/MS spectra analysis we also noted that rather large peptides (>2500 Da) revealed in many cases improved sequence fragmentation when detected by MALDI-TOF/TOF, compared to the same peptide analyzed by ESI in the linear trap. The use of ETD (electron transfer dissociation) [64] as a fragmentation technique of the tryptic peptides had only a minor impact on the improvement of the sequence coverage of the proteins investigated. However, triply charged precursor ions fragmented by ETD yielded (in some cases) more complete series of product ions and therefore more extensive sequence information when compared with the corresponding CID spectra. It is also important to note that the database search of the MS/MS spectra revealed substantially more positive results when semi-tryptic sequences were considered compared to the fully tryptic approach (dual tryptic termini). However, database searches including no specification of the enzyme did not improve protein identifications. This could possibly be explained as a significant increase in the search space, which ultimately reduces the search engine’s sensitivity [65].

## Conclusions

The combined approach of electrospray and MALDI mass spectrometry increased the sequence coverage of the predicted protein families (metalloproteinases, serine-proteinases, CRISP, C-type lectin, L-amino acid oxidase, vascular endothelial growth factor, bradykinin-potentiating protein and phospholipase A_2_) and their corresponding isoforms when compared to one ionization technique alone. Additionally, this approach also revealed the presence of snake venom protein families (glutaminyl cyclase, renin-like aspartic protease and ecto 5'-nucleotidase) previously not encountered in the transcriptome of the venom gland of *S*.*c edwardsii*. These results support the use of a dual technical approach toward determining the proteome of venoms, which have both abundant and rare protein components, in order to obtain a more complete analysis. Our results revealed increased diversity of venom constituents in this venom and provide support for future studies of structure-function relationships of several venom protein family isoforms.

## Supporting Information

S1 Table Sequences of venom proteins from *S*. *c*. *edwardsii* identified by tandem mass spectrometry. Search engine color code for the identified peptides: Peaks white, ProLucid grey, and Mascot light blue.
(DOC)

S2 Table Comparison of the sequences determined by *de novo* sequencing of MS/MS spectra of the venom of *S*. *edwardsii* (Sanz *et al*.) with the sequences detected in the present study. (DOC)

S3 Table Comparison of the sequences determined by Edmann sequencing of the venom of *S*. *edwardsii* (Sanz *et al*.) with the sequences detected in the present study. (DOC)

S4 Table Sequence coverages of some of the predicted venom gland proteins of *S*. *c*. *edwardsii* as revealed by the combined approach of MALDI and ESI tandem mass spectrometry. Protein sequences including specific domains are indicted by colored bars; below these, corresponding peptides identified by ESI (black lines) and MALDI (red lines) are indicated (Part 1).
(DOCX)

S5 Table Sequence coverages of some of the predicted venom gland proteins of *S*. *c*. *edwardsii* as revealed by the combined approach of MALDI and ESI tandem mass spectrometry. Protein sequences including specific domains are indicted by colored bars; below these, corresponding peptides identified by ESI (black lines) and MALDI (red lines) are indicated (Part 2).
(DOCX)
