# Supplementary material for: Interrogating the Venom of the Viperid Snake Sistrurus catenatus edwardsii by a Combined Approach of Electrospray and MALDI Mass Spectrometry
Source: PLoS One. 2015 May 8;10(5):e0092091. doi: 10.1371/journal.pone.0092091 (PMC4425365; doi:10.1371/journal.pone.0092091)
Supplement: S3 Table — (DOC) [file pone.0092091.s003.doc]

Chapeaurouge et al., Supplemental Table 3
