# Supplementary material for: Interrogating the Venom of the Viperid Snake Sistrurus catenatus edwardsii by a Combined Approach of Electrospray and MALDI Mass Spectrometry
Source: PLoS One. 2015 May 8;10(5):e0092091. doi: 10.1371/journal.pone.0092091 (PMC4425365; doi:10.1371/journal.pone.0092091)
Supplement: S4 Table — Protein sequences including specific domains are indicted by colored bars; below these, corresponding peptides identified by ESI (black lines) and MALDI (red lines) are indicated (Part 1). (DOCX) [file pone.0092091.s004.docx]

| Protein  (Coverage %) | Sequence |
| --- | --- |
| Serine proteinase 1  (81.6) |  |
| Serine proteinase 2  (76) |  |
| Serine proteinase 3  (72.5) |  |
| Serine proteinase 4  (72) |  |
| Serine proteinase 5  (48.6) |  |
| Serine proteinase 6  (37.8) |  |
| Serine proteinase 7  (56.4) |  |
| Serine proteinase 8  (35.2) |  |
| Serine proteinase 9  (37.9) |  |
