# Supplementary material for: Interrogating the Venom of the Viperid Snake Sistrurus catenatus edwardsii by a Combined Approach of Electrospray and MALDI Mass Spectrometry
Source: PLoS One. 2015 May 8;10(5):e0092091. doi: 10.1371/journal.pone.0092091 (PMC4425365; doi:10.1371/journal.pone.0092091)
Supplement: S5 Table — Protein sequences including specific domains are indicted by colored bars; below these, corresponding peptides identified by ESI (black lines) and MALDI (red lines) are indicated (Part 2). (DOCX) [file pone.0092091.s005.docx]

| Protein  (Coverage %) | Sequence |
| --- | --- |
| Serine proteinase 10  (51) |  |
| Serine proteinase 11  (44.2) |  |
| C-type lectin 1  (74.7) |  |
| C-type lectin 2  (28.5) |  |
| C-type lectin 3  (35.9) |  |
| L-amino acid oxidase  (65) |  |
| CRISP  Isoform 2 (59.4) |  |
| Vascular endothelial growth factor isoform 2  (43.9) |  |
