# Supplementary material for: Interrogating the Venom of the Viperid Snake Sistrurus catenatus edwardsii by a Combined Approach of Electrospray and MALDI Mass Spectrometry
Source: PLoS One. 2015 May 8;10(5):e0092091. doi: 10.1371/journal.pone.0092091 (PMC4425365; doi:10.1371/journal.pone.0092091)
Supplement: Supplementary file 5 [file pone.0092091.s001.doc]

Protein (gi)	Sequence	m/z	score
metalloproteinase proteoform 1 109254960	VTDLLR	358,72	37.47
	QAYLDAK	404,72	37.87
	GYCYNR	416,68	41.23
	TYLINSR	434,24	33.05
	FGPSATVAK	439,25	52.58
	YKDDLDK	448,73	52.25
	GSSYGYCR	475,2	55.61
	LTPGSQCAK	481,25	40.14
	IYIPLNIR	501,32	35.64
	GLCCDQCR	506,2	45.23
	LTPGSQCAKG	509,76	36.28
	LFCFPNKP	511,77	30.82
	FFGPSATVAK	512,78	45.45
	FKGAGTECR	513,25	56.81
	LFCFPNKPG	540,28	48.93
	GHCVDVATAY	546,75	34.51
	EIYIPLNIR	565,84	36.75
	NGHCVDVATAY	603,77	42.88
	LFCFPNKPGK	604,33	59.90
	NEIYIPLNIR	622,86	34.46
	NQCISFFGPSAT	664,81	43.82
	GSVGVIQEHSTIN	670,85	52.31
	NLTPEQQAYLDAK	745,89	47.02
	HQPSTQFSDCSEK	775,84	55.21
	GSVGVIQEHSTINLL	783,94	49.69
	LSHQPSTQFSDCSEK	875,9	76.14
	SQLNLTPEQQAYLDAK	909,98	60.20
	VTDLLR	716,44	0.37
	GSVGVIQEH	925,48	0.49
	GSSYGYCR	949,39	0.42
	LFCFPNKP	1022,52	0.34
	LFCFPNKPG	1079,55	0.45
	LFCFPNKPG	1079,55	0.45
	STQFSDCSEK	1188,5	0.43
	LSHQPSTQFSDC	1406,62	0.5
	HQPSTQFSDCSEK	1550,67	0.48
	NQCISFFGPSATVAK	1626,82	0.55
	NQCISFFGPSATVAK	1628,8	0.56
	LSHQPSTQFSDCSEK	1750,79	0.54
	PQCILNEPLLTDIVSPPVCGN	2337,2	0.52
	SHDNAQLLTTTDFDGDTVGLAY	2355,11	0.51
	ELLEEGEECDCGSPANCQNPCCDAATCK	3275,26	0.52
	TYLINSR	866,47	39
	GSSYGYCR	949,39	38
	LTPGSQCAK	961,49	60
	NGHPCLNNK	1053,45	66

metalloproteinase proteoform 1 109254960	GLCCDQCR	1068,43	36
	LFCFPNKPGK	1207,64	63
	NQCISFFGPSATVAK	1626,8	77
	SHQPSTQFSDCSEK	1637,73	91
	QLNLTPEQQAYLDAK	1714,87	97
	LSHQPSTQFSDCSEK	1750,8	103
	TTTDFDGDTVGLAYMR	1762,81	92
	YVEFVVVLDHGMYTK	1799,87	100
	IYEIVNTMNEIYIPLNIR	2208,14	119
	NICNVIYTPTDEDIGMVLPGTK	2450,15	89
	SHDNAQLLTTTDFDGDTVGLAYMR	2641,28	146
	EMGHNLGMSHDGNQCHCGAPSCIMADR	3042,2	88
	ELLEEGEECDCGSPANCQNPCCDAATCK	3274,36	111
metalloproteinase proteoform 2 109254962	SFGEWR	391,19	36.98
	HLNNFR	401,21	48.58
	HLNNFR	401,21	46.38
	VIVADYR	418,24	35.08
	ILNEPLR	427,76	40.22
	YYGYCR	441,19	43.67
	KPQCILNEPLR	456,59	31.28
	GNYYGYCR	498,21	33.00
	CILNEPLR	507,78	40.88
	NTLNEIYR	511,77	41.85
	TWVYEIVN	512,27	18.98
	FKGAGTECR	513,25	56.81
	IPCAPEDVK	514,76	40.90
	STGIVQDHSP	520,76	54.33
	FYKPQCILNEPLR	559,97	34.91
	QDSCFDDNR	578,73	56.14
	KIPCAPEDVK	578,81	46.10
	IVNTLNEIYR	617,85	58.09
	PQCILNEPLR	620,34	53.72
	QIPCVPYYTR	648,83	42.75
	LHSWVECESGE	666,79	48.20
	EIVNTLNEIYR	673,37	22.96
	EAQDSCFDDNR	678,77	43.82
	ADDTLDSFGEWR	706,2	62.72
	AEAQDSCFDDNR	714,29	59.62
	TWVYEIVNTLNE	740,88	30.95
	AAEAQDSCFDDNR	749,81	66.39
	SADDTLDSFGEWR	749,84	67.26
	YKPQCILNEPLR	765,92	54.61
	LSADDTLDSFGEWR	806,38	72.07
	VYEIVNTLNEIYR	813,44	68.16
	AASSECDLPENCTGH	824,33	58.24
	TWVYEIVNTLNEIYR	957,01	74.30

metalloproteinase proteoform 2 109254962	STGIVQDHSP	1040,51	0.45
	QDSCFDDNR	1156,44	0.36
	IGNYYGYCR	1165,52	0.56
	IGNYYGYCR	1166,51	0.43
	IGNYYGYCR	1166,51	0.39
	AEAQDSCFDDNR	1427,57	0.54
	AAEAQDSCFDDNR	1498,6	0.59
	AASSECDLPENCTGH	1647,66	0.54
	FYKPQCILNEPLR	1677,9	0.47
	NFNGNQIPCVPYYTR	1844,87	0.52
	FGPNAAEAQDSCFDDNR	1940,8	0.63
	YNFNGNQIPCVPYYTR	2007,94	0.45
	YNFNGNQIPCVPYYTR	2007,94	0.48
	LFGPNAAEAQDSCFDDNR	2027,89	0.63
	SYNFNGNQIPCVPYYTR	2094,97	0.51
	ALFGPNAAEAQDSCFDDNR	2098,92	0.61
	LYCSYNFNGNQIPCVPY	2110,94	0.35
	LYCSYNFNGNQIPCVPY	2110,94	0.31
	CSYNFNGNQIPCVPYYTR	2255,01	0.43
	YALFGPNAAEAQDSCFDDNR	2261,99	0.59
	LYCSYNFNGNQIPCVPYYT	2376,06	0.36
	YCSYNFNGNQIPCVPYYTR	2418,07	0.46
	LYCSYNFNGNQIPCVPYYTR	2531,17	0.46
	KIPCAPED	1156,64	57
	DENMGMVEPGTK	1290,63	90
	YIELVIVADYR	1353,71	65
	RDENMGMVEPGTK	1446,74	77
	AASSECDLPENCTGH	1647,68	102
	FGPNAAEAQDSCFDDNR	1913,77	105
	ALFGPNAAEAQDSCFDDNR	2097,91	152
	YALFGPNAAEAQDSCFDDNR	2260,98	162
	YALFGPNAAEAQDSCFDDNR	2260,98	151
	HQCYALFGPNAAEAQDSCFDDNR	2686,13	71
	STGIVQDHSPINLLVAVTMAHEMGHNLGIR	3210,63	182
metalloproteinase proteoform 3 109254964	SIDFDGR	405,2	37.95
	ITVQSSAH	421,73	31.73
	TVGLAHVSS	435,74	44.63
	TSIDFDGR	455,72	57.88
	LSTGVIQDH	485,26	47.78
	IYIPLNIR	501,32	35.64
	FKGAGTECR	513,25	56.81
	QCVDVTTAY	528,74	59.20
	LEIWSNGDK	531,27	26.27
	LFCEFGHY	536,74	46.27
	DACFDFNLK	565,26	33.84
	EIYIPLNIR	565,84	36.75
	HDNAQLLTSIDFDGR	568,28	40.15
	LLTSIDFDGR	568,81	55.62

metalloproteinase proteoform 3 109254964	SCTGQSADCPTDDLHR	607,26	62.29
	VGLEIWSNGDK	609,32	54.57
	NEIYIPLNIR	622,86	34.46
	QLLTSIDFDGR	632,84	59.29
	ITVQSSAHNTLH	654,35	61.06
	LVGLEIWSNGDK	665,86	56.74
	VAPDACFDFNLK	698,84	64.07
	LFCEFGHYSCQ	724,3	58.52
	TINEIYIPLNIR	730,42	34.27
	FALVGLEIWSNGDK	774,92	71.92
	FALVGLEIWSNGDK	775,41	62.25
	VNTINEIYIPLNIR	836,49	47.82
	HDNAQLLTSIDFDGR	851,43	80.96
	CTGQSADCPTDDLHR	866,87	65.32
	GQDNFYCR	1059,44	0.5
	DACFDFNLK	1129,51	0.30
	LLTSIDFDGR	1136,61	0.46
	CDCGSPNTCR	1226,45	0.44
	LFCEFGHYSCQ	1447,59	0.56
	FALVGLEIWSNGDK	1548,83	0.58
	FALVGLEIWSNGDK	1549,81	0.54
	VGEECDCGSPNTCR	1640,63	0.57
	ESGNVNDYEVVYPR	1641,78	0.49
	HDNAQLLTSIDFDGR	1701,84	0.66
	HDNAQLLTSIDFDGR	1702,82	0.44
	CTGQSADCPTDDLHR	1733,72	0.56
	EVGEECDCGSPNTCR	1769,68	0.57
	QHSWVECESGECCE	1797,66	0.63
	GADATVAPDACFDFNLK	1812,85	0.47
	SCTGQSADCPTDDLHR	1819,75	0.49
	SCTGQSADCPTDDLHR	1820,76	0.59
	LEVGEECDCGSPNTCR	1882,76	0.59
	YGADATVAPDACFDFNLK	1975,92	0.58
	LLEVGEECDCGSPNTCR	1996,85	0.65
	ELLEVGEECDCGSPNTCR	2125,89	0.62
	ELLEVGEECDCGSPNTCR	2126,88	0.62
	ALYGADATVAPDACFDFNLK	2160,05	0.52
	QHSWVECESGECCEQCK	2212,84	0.57
	QCIALYGADATVAPDACFDFNLK	2562,23	0.52
	SECDIAESCTGQSADCPTDDLHR	2625,07	0.61
	AHVSSMCNQK	1161,53	53
	PDFGMVDHGTK	1203,52	86
	YVELVIVADHGM	1345,7	85
	ECDCGSPNTCR	1355,5	83
	SGDPDFGMVDHGTK	1462,62	105
	SSAHNTLHLFGNWR	1639,83	62
	KKHDNAQLLTSIDFDGR	1958,03	123

metalloproteinase proteoform 3 109254964	ITVQSSAHNTLHLFGNWR	2081,03	90
	HSWVECESGECCEQCK	2084,84	86
	QHSWVECESGECCEQCK	2195,75	138
	TWVFQMVNTINEIYIPLNIR	2464,31	119
	SECDIAESCTGQSADCPTDDLHR	2624,11	223
	LSTGVIQDHSAINLVMAVTMAHELGH	2744,41	77
	FCEFGHYSCQYQYSGDPDFGMVDHGTK	3232,33	100
	TDIVSPPVCGNELLEVGEECDCGSPNTCR	3264,42	182
metalloproteinase proteoform 5 109254968	SADCPTDDFQR	656,28	35.25
	CTGQSADCPTDDFQR	879,36	58.07
	LQGQGDFYCR	622,29	50.52
	NLQGQGDFYCR	679,31	53.06
	NYNLQGQGDFYCR	817,87	61.89
	CFNYNLQGQGDFYCR	971,42	69.83
	FPCAPQDVK	1061,52	0.42
	LQGQGDFYCR	1243,56	0.42
	NYNLQGQGDFYCR	1634,72	0.47
	NGQPCLNNNGYCYNGK	1874,77	0.44
	CFNYNLQGQGDFYCR	1941,83	0.59
	SECDIAESCTGQSADCPTDDFQR	2650.05	0.59
	NNNGYCYNGK	1203,46	81
	GKPGTQCEDGECCDR	1824,78	29
	NGQPCLNNNGYCYNGK	1872,79	83
	TISCQSTYSQNDLDIGMVDLGTK	2546,2	59
	SECDIAESCTGQSADCPTDDFQR	2649,01	214
metalloproteinase proteoform 6 109254970	ETVLLNR	422,75	40.73
	LEAFGDWR	497,25	48.39
	GLCCDQCR	506,2	45.23
	IELVVVADHR	575,84	63.66
	CTGQSADCPR	576,24	74.48
	DTLEAFGDWR	605,29	59.28
	AADTLEAFGDWR	676,33	63.87
	AAADTLEAFGDWR	711,85	65.15
	SAAADTLEAFGDWR	755,36	64.08
	NVQSAAADTLEAFGDWR	925,95	81.16
	LEAFGDWR	993,49	0.43
	LSTGIVQDHSA	1127,58	0.43
	CTGQSADCPR	1151,47	0.48
	DTLEAFGDWR	1209,57	0.49
	AAADTLEAFGDWR	1422,68	0.56
	CAEGLCCDQCR	1428,53	0.49
	SAAADTLEAFGDWR	1509,72	0.61
	AQCAEGLCCDQCR	1627,63	0.43
	LRPGAQCAEGLCCDQC	1895,79	0.58
	INVQSAAADTLEAFGDWR	1964,98	0.66

metalloproteinase proteoform 6 109254970	INVQSAAADTLEAFGDWR	1964,98	0.52
	LRPGAQCAEGLCCDQCR	2050,89	0.58
	ECDCGSPANPCCDAATCK	2073,75	0.62
	EECDCGSPANPCCDAATCK	2202,8	0.58
	GEECDCGSPANPCCDAATCK	2259,82	0.52
	QDQINVQSAAADTLEAFGDWR	2336,14	0.60
	EAGEECDCGSPANPCCDAATCK	2460,91	0.61
	YNSNLNTVR	1080,57	67
	AAADTLEAFGDWR	1422,63	59
	SAAADTLEAFGDWR	1509,68	71
	IWVHEIVNIINVFYR	1915,04	74
	LRPGAQCAEGLCCDQCR	2050,82	77
	GEECDCGSPANPCCDAATCK	2258,73	148
	TAIELDEETIGLAHVGSMCDPK	2386,16	51
	EAGEECDCGSPANPCCDAATCK	2458,97	146
metalloproteinase proteoform 7 109254972	GLEIWSNR	487,76	38.32
	GLCCDQCR	506,2	45.23
	FKGAGTECR	513,25	56.81
	ECIDSFQR	527,74	37.62
	IACEPQDVK	530,27	49.60
	VGLEIWSNR	537,3	51.57
	TDVVSPAVCGN	559,77	54.43
	LVGLEIWSNR	593,84	68.86
	SAECIDSFQR	606,78	64.59
	YCFPNSPETK	621,78	49.41
	SQSNLTPEQQR	644,33	48.95
	SSPDVTLDLFAK	646,85	67.51
	LYCFPNSPETK	678,33	65.22
	VSQDACFQFNR	686,32	54.52
	ACSNGQCVDVTTPY	786,34	54.02
	CEASFLFSDCSQK	789,84	60.92
	PCNIYYSPNDEDK	807,85	59.38
	IVQSSPDVTLDLFAK	816,96	62.29
	FVEVGEECDCGSPR	820,85	70.33
	NPCNIYYSPNDEDKG	893,38	64.00
	YFVEVGEECDCGSPR	902,38	75.90
	IACEPQDVK	1059,52	0.46
	DPCCDAATCK	1198,54	0.54
	SAECIDSFQR	1212,54	0.47
	SAECIDSFQR	1212,55	0.54
	ETCTCGTRPC	1241,48	0.45
	IYYSPNDEDK	1243,56	0.39
	CEASFLFSDCSQK	1278,68	0.53
	SSPDVTLDLFAK	1292,69	0.43
	LYCFPNSPETK	1355,65	0.48
	VALVGLEIWSNR	1356,78	0.45

metalloproteinase proteoform 7 109254972	VSQDACFQFNR	1371,63	0.45
	LYCFPNSPETKN	1469,69	0.40
	AQCAEGLCCDQCR	1627,63	0.43
	IVQSSPDVTLDLFAK	1632,91	0.54
	IIVQSSPDVTLDLFAK	1747	0.52
	YFVEVGEECDCGSPR	1803,76	0.62
	QGAQCAEGLCCDQCR	1812,71	0.53
	FGPGATVSQDACFQFNR	1902,89	0.6
	SHDNAQLLTGINFNGPTAG	1927,96	0.58
	AGTLSCEASFLFSDCSQK	2008,91	0.58
	CIALFGPGATVSQDACFQFNR	2360,13	0.63
	MYDTVNVITPIYHR	1721,87	100
	NPCNIYYSPNDEDK	1728,76	78
	YFVEVGEECDCGSPR	1803,73	118
	QGAQCAEGLCCDQCR	1812,7	119
	MNIHVALVGLEIWSNR	1851,98	93
	MNIHVALVGLEIWSNRDK	2095,11	57
	NPCNIYYSPNDEDKGMVLPGTK	2512,17	55
	IHHLVAIAMAHEMGHNLGMDHDK	2577,27	166
serine proteinase proteoform 1 109254938	LDRPVS	343,7	33.17
	AGILEGGK	372,72	31.63
	DIMLIR	380,73	39.93
	LDRPVSN	400,72	49.30
	FLVALYH	431,75	26.72
	FFCLSSK	444,72	53.10
	CAGILEGGK	452,74	50.00
	ETYPNVPH	478,73	39.11
	LDRPVSNSE	508,76	53.70
	LDRPVSNSE	509,25	29.34
	LCAGILEGGK	509,28	53.46
	IIAGNTDATCPP	615,3	59.06
	GGDECNINEHR	650,78	63.57
	ETYPNVPHCAN	651,29	58.47
	SLPSSPPSVGSVCR	715,37	56.30
	ETYPNVPHCANIN	764,86	47.77
	LSLPSSPPSVGSVCR	771,91	65.73
	TLCAGILEGGK	1118,6	0.46
	ETYPNVPHCAN	1301,57	0.36
	TFLCGGTLLNEEW	1540,74	0.56
	GDSGGPLICNGQFQGILSWGDDPCGY	2773,23	0.48
	FLVALYHSR	1105,62	62
	TVPNEDEQTR	1188,6	44
	LDRPVSNSEHIAPL	1247,82	91
	SLPSSPPSVGSVCR	1429,71	113
	VAAYPEYMLPATSR	1568,78	67
	VAAYPEYMLPATSR	1568,78	67

serine proteinase proteoform 1 109254938	SEHIAPLSLPSSPPSVGSVCR	2177,13	97
	VFDHLDWIQSIIAGNTDATCPP	2470,17	174
	TFLCGGTLLNEEWVLTAAHCD	2563,18	73
	INILNYEVCVAAYPEYMLPATSR	2687,32	51
	CANINILNYEVCVAAYPEYMLPATSR	3032,41	53
serine proteinase proteoform 2 109254940	AGILEGGK	372,72	31.63
	DIMLIR	380,73	39.93
	EPGLYTK	404,22	45.78
	CAGILEGGK	452,74	50.00
	EYGLPATSR	497,26	43.64
	LCAGILEGGK	509,28	53.46
	LLNYSVCR	512,77	39.04
	PEYGLPATSR	545,79	52.50
	IIAGNTDATCPP	615,3	59.06
	YPEYGLPATSR	627,32	52.77
	VFDHLDWIQS	630,31	19.18
	SWGSPNCGYVR	641,79	39.82
	GGDECNINEHR	650,78	63.57
	AYPEYGLPATSR	662,84	55.84
	AAYPEYGLPATSR	698,36	65.45
	LSLPSSPPSVGSVCR	771,91	65.73
	FFCVSSK	874,42	0.35
	TLCAGILEGGK	1119,56	0.44
	IIGGDECNINEHR	1526,72	0.53
	VILPDVPHCVNINLLN	1830,02	0.43
	DTCVGDSGGPLICNGQFQGIA	2167,98	0.56
	DTCVGDSGGPLICNGQFQGIASWGSPN	2795,26	0.55
	VYSDGNQCGGTLINEEWVLTAAHCEGNK	3124,44	0.55
	LVYSDGNQCGGTLINEEWVLTAAHCEGNK	3236,53	0.52
	MGWGTISPTK	1077,53	88
	WDKDIMLIR	1189,63	65
	AAYPEYGLPATSR	1395,68	100
	LDRPVGNSEHIAPL	1517,8	66
	IIGGDECNINEHR	1526,72	113
	IAPLSLPSSPPSVGSVCR	1542,78	130
	IAPLSLPSSPPSVGSVCR	1823,95	131
	SEHIAPLSLPSSPPSVGSVCR	2177,09	114
	VFDHLDWIQSIIAGNTDATCPP	2470,17	174
	VILPDVPHCVNINLLNYSVCR	2495,28	83
	LDRPVGNSEHIAPLSLPSSPPSVGSVCR	2928,51	102
	VYSDGNQCGGTLINEEWVLTAAHCEGNK	3122,52	140
	TCVGDSGGPLICNGQFQGIASWGSPNCGY	3429,56	169

serine proteinase isoform 3 109254942	LKPGVYTK	302,52	40.96
	AGILEGGK	372,72	31.63
	FFCPDK	407,19	50.84
	CAGILEGGK	452,74	50.00
	IGGDECNVNEHR	467,21	21.44
	FFCPDKK	471,24	54.12
	HIIGWGSITPIEK	484,28	36.14
	ALKPGVYTK	488,8	41.48
	LCAGILEGGK	509,28	53.46
	TLPDVPYCAN	575,27	30.23
	IIAGNTAATCPP	593,31	42.70
	VFDYNDWIQ	600,28	24.27
	IGWGSITPIEK	600,84	54.86
	VFDYNDWIQS	643,8	46.77
	LDSPVSNSEHIAPL	739,89	38.96
	VIGGDECNVNEHR	749,85	64.24
	EWVLTAAHCDSTNF	825,88	56.93
	LLDDAVCQPPYPELPATSR	1071,55	74.97
	ANINLLDDAVCQPPYPELPATSR	1277,65	76.62
	FFCPDKK	941,46	0.43
	FCAGTLINK	1023,54	0.3
	LDSPVSNSEH	1084,5	0.43
	LDSPVSNSEH	1084,5	0.43
	TLCAGILEGGK	1118,6	0.46
	VFDYNDWIQS	1286,58	0.32
	IIGWGSITPIEK	1313,76	0.55
	LDSPVSNSEHIAP	1365,68	0.49
	LDSPVSNSEHIAPL	1478,77	0.39
	EWVLTAAHCDSTNF	1650,74	0.51
	LLDDAVCQPPYPELPATSR	2142,08	0.55
	DTCGGDSGGPLICNGQFQGIVF	2300,05	0.59
	DTCGGDSGGPLICNGQFQGIVFY	2464,11	0.49
	DTCGGDSGGPLICNGQFQGIVFYGAHPCG	3043,34	0.49
	FFCPDKK	941,43	39
	IIGWGSITPIEK	1313,68	72
	DDVLDKDIMLIK	1417,77	102
	KDDVLDKDIMLIK	1545,87	95
	YGAHPCGQALKPGVYTK	1846,99	101
	INLLDDAVCQPPYPELPATSR	2369,24	168
	TLPDVPYCANINLLDDAVCQPPYPELPATSR	3499,69	132

serine proteinase proteoform 4	SLVVFF	711,42	29.0
	LDRPVS	343,7	33.17
	AGILEGGK	372,72	31.63
	DIMLIR	380,73	39.93
	LDRPVSN	400,72	49.30
	KVLNEDEQTR	411,22	37.03
	TSVGSVCR	433,22	42.46
	CAGILEGGK	452,74	50.00
	GWGINSPTK	480,26	41.27
	LDRPVSNSE	508,76	53.70
	LDRPVSNSE	509,25	29.34
	LCAGILEGGK	509,28	53.46
	VLNEDEQTR	552,28	54.25
	IIAGNTDATCPP	615,3	59.06
	IIAGNTDATCPP	615,31	59.06
	SGTLCAGILEGGK	631,83	62.10
	ETYPDVPHCAN	651,78	59.24
	SIIAGNTDATCPP	658,82	46.70
	ETYPDVPHCANI	680,31	20.80
	TSGTLCAGILEGGK	682,36	60.76
	AGTLINQEWVLTAAHCDSK	705,36	56.62
	VGGEECNINEHR	707,32	53.01
	SLPSSPTSVGSVCR	717,37	63.34
	VVGGEECNINEHR	756,86	52.93
	LSLPSSPTSVGSVCR	773,91	60.59
	SLTATSGTLCAGILEGGK	868,46	71.66
	IAPLSLPSSPTSVGSVCR	914,51	67.79
	AAYPSLTATSGTLCAGILEGGK	1069,55	72.83
	KVLNEDEQTR	1231.64	0.49
	ETYPDVPHCANIN	1530.69	0.41
	LDRPVSNSE	1016,51	0.5
	LDRPVSNSEH	1153,57	0.41
	TATSGTLCAGILEGGK	1536,81	0.54
	SLTATSGTLCAGILEGGK	1735,91	0.57
	DACGGDSGGPLICNGEIQG	1877,81	0.57
	AAYPSLTATSGTLCAGILEGGK	2140,12	0.61
	QAAYPSLTATSGTLCAGILEGGK	2267,17	0.56
	DACGGDSGGPLICNGEIQGIVSGGEHPCG	2872,26	0.58
	LDRPVSNSEHIAPLSLPSSPTSVGSVCR	2963,55	0.54
	DKDIMLIR	1003,56	61
	IMGWGINSPTK	1203,59	81
	NFQMQLGVHSK	1288,64	88
	VVGGEECNINEHR	1512,67	106
	LSLPSSPTSVGSVCR	1546,76	121
	ETYPDVPHCANINILH	1892,8	85

serine proteinase proteoform 4	ETYPDVPHCANINILHHEVCQ	2546,14	94
	DACGGDSGGPLICNGEIQGIVSGGEHPCG	2870,24	125
	LDRPVSNSEHIAPLSLPSSPTSVGSVCR	2962,58	101
	INILHHEVCQAAYPSLTATSGTLCAGILEGGK	3381,74	54
serine proteinase proteoform 5	AGILEGGK	372,72	31.63
	EPGVYTK	397,21	44.52
	CAGILEGGK	452,74	50.00
	LCAGILEGGK	509,28	53.46
	IIAGNTDATCPP	615,3	59.06
	GGDECNINEHR	650,78	63.57
	VSWGGDICAQPR	673,33	59.24
	IVSWGGDICAQPR	729,87	63.72
	VIGGDECNINEHR	756,86	58.62
	GIVSWGGDICAQPR	758,38	59.03
	TLCAGILEGGK	1118,6	0,45
	IIAGNTDATCPP	1229,6	0,46
	VIGGDECNINEHR	1512,7	0,47
	GIVSWGGDICAQPR	1515,76	0,52
	SSCDGDSGGPLICNGEIQG	1923,82	0,52
	SSCDGDSGGPLICNGEIQGIVSWGGDICAQPR	3364,54	0.50
	FICPNR	806,4	32
	DKDIMLIR	1003,56	58
	AAYPSLTATSR	1137,56	68
	NDEKDKDIMLIR	1489,76	98
	ILDHEVCQAAYPSLTATSR	2132,05	103
	CANINILDHEVCQAAYPSLTATSR	2704,32	81
	SSCDGDSGGPLICNGEIQGIVSWGGDICAQPR	3362,69	82
serine proteinase proteoform 6 109254948	DIMLIR	380,73	39.93
	FLVALYH	431,75	26.72
	LLDYEVCR	534,27	44.03
	GGDECNINEHR	650,78	63.57
	SLPSSPPSVGSVCR	715,37	56.30
	ANINLLDYEVCR	740,38	58.58
	VIGGDECNINEHR	756,86	58.62
	LSLPSSPPSVGSVCR	771,91	65.73
	LNKPVSY	820,46	0,39
	VFDHLDW	931,44	0,38
	PSSPPSVGSVCR	1230,61	0,48
	TFLCGGTLLNEEW	1539,74	0,56
	TFLCGGTLLNEEWVLT	1853,95	0,48
	DKDIMLIR	1003,56	58
	FLVALYHSR	1105,59	51
	FLVALYHSR	1105,62	62
	IAPLSLPSSPPSVGSVCR	1823,95	131
	SEHIAPLSLPSSPPSVGSVCR	2177,09	114
	TFLCGGTLLNEEWVLTAAHCNR	2563,18	76

serine proteinase isoform 6 109254948	VIGGDECNINEHR	756,86	58.62
	LSLPSSPPSVGSVCR	771,91	65.73
	LNKPVSY	820,46	0,39
	VFDHLDW	931,44	0,38
	PSSPPSVGSVCR	1230,61	0,48
	TFLCGGTLLNEEW	1539,74	0,56
	TFLCGGTLLNEEWVLT	1853,95	0,48
	DKDIMLIR	1003,56	58
	FLVALYHSR	1105,59	51
	FLVALYHSR	1105,62	62
	IAPLSLPSSPPSVGSVCR	1823,95	131
	SEHIAPLSLPSSPPSVGSVCR	2177,09	114
	TFLCGGTLLNEEWVLTAAHCNR	2563,18	76
serine proteinase proteoform 7 109254950	SEHIAP	327,17	16.44
	AGILEGGK	372,72	31.63
	DIMLIR	380,73	39.93
	SEHIAPL	383,71	18.38
	CAGILEGGK	452,74	50.00
	LCAGILEGGK	509,28	53.46
	AAYGGSPATSR	519,26	50.00
	SSPPSVGSVCR	5667,8	52.21
	IISGNTDATCPP	623,3	41.33
	SLPSSPPSVGSVCR	715,37	56.30
	IVSWGADTCAQPR	730,86	45.97
	VIGGDECNVNEHR	749,85	64.24
	LSLPSSPPSVGSVCR	771,91	65.73
	QGIVSWGADTCAQPR	823,4	69.26
	GQFQGIVSWGADTCAQPR	989,48	43.38
	CAGILEGGK	904,46	0,49
	YFCLSSR	932,44	0,32
	AAYGGSPATSR	1037,51	0.32
	TLCAGILEGGK	1118,6	0,37
	IVSWGADTCAQPR	1461,72	0.41
	GQFQGIVSWGADTCAQPR	1977,96	0.37
	APGFYTNVFDYTDWIQS	2024,94	0,55
	FQCGGTLINEEWVLTAAH	2047,01	0,64
	YFCLSSR	932,41	32
	GDKDIMLIR	1060,58	57
	VIGGDECNVNEHR	1498,7	84
	SEHIAPLSLPSSPPSVGSVCR	2177,09	114
	INILDYEVCQAAYGGSPATSR	2285,11	94

serine proteinase proteoform 8 109254952	CAGILEGGK	452,74	50.00
	EIYPNVPR	494,27	42.68
	LCAGILEGGK	509,28	53.46
	ILCAGILEGGK	565,82	65.72
	INILDYAVCR	618,83	41.30
	VFDYIDWIQS	643,32	42.61
	LSLPSSPPSVGSLCR	778,92	60.05
	LNPGFYTK	939,5	0,26
	ILCAGILEGGK	1130,64	0.48
	INILDYAVCR	1236,66	0.52
	AAYPWWPVTTR	1347,7	0.36
	YPWWPVTTR	1205,62	42
	AAYPWWPVTTR	1347,71	31
serine proteinase proteoform 9 109254956	AGILEGGK	372,72	31.63
	CAGILEGGK	452,74	50.00
	CAQPDKPSLYTK	469,91	46.07
	LCAGILEGGK	509,28	53.46
	VTNEDVQTR	531,27	63.15
	FFCLSSK	888,44	0.22
	CAGILEGGK	904,46	0.49
	TLCAGILEGGK	1119,56	0.44
	CAQPDKPSLYTK	1407,71	0.53
	FFCLSSK	888,43	41
	DKDIMLIR	1003,56	58
	VTNEDVQTR	1061,54	46
	TLCAGILEGGK	1118,64	96
	VFDHLDWIQSIIAGNK	1855,95	98
	WGPDTCAQPDKPSLYTK	1963,92	97
	ILDHEVCQAAYPSLTATSR	2132,05	103
	INILDHEVCQAAYPSLTATSR	2359,21	103
	CANINILDHEVCQAAYPSLTATSR	2704,32	81
serine proteinase proteoform 10 109254954	DIMLIR	380,73	39.93
	FLVALYH	431,75	26.72
	YSSLPATSR	491,26	43.79
	LLDYEVCR	534,27	44.03
	AAYSSLPATSR	562,3	52.14
	ANINLLDYEVCR	740,38	58.58
	IIGGEECNINEHR	770,87	65.41
	LSLPSSPPSVGSVCR	771,91	65.73
	VLCAGVLEGGIDTCNR	867,43	88.62
	NVIFDDDHIR	415,21	86.75
	DKDIMLIR	1002,55	22.24
	LNKPVS	820,46	0.39
	VFDHLDW	931,44	0.38
	NVIFDDDHIR	1243,62	0.41
	TFLCGGTLLNEEW	1539,74	0.5

serine proteinase proteoform 10 109254954	IIGGEECNINEHR	1540,73	0.58
	TFLCGGTLLNEEWVLT	1853,95	0.48
	VFMYIR	828,44	33
	FLVALYHSR	1105,62	73
	AAYSSLPATSR	1123,6	55
	WDKDIMLIR	1189,65	70
	IAPLSLPSSPPSVGSVCR	1823,95	131
	TFLCGGTLLNEEWVLTAAHCNR	2563,18	76
	LNKPVSYSEYIAPLSLPSSPPSVGSVCR	3004,58	101
serine proteinase proteoform 11 109254958	AGILEGGK	372,72	31.63
	DIMLIR	380,73	39.93
	FFCLSSK	444,72	53.10
	CAGILEGGK	452,74	50.00
	LCAGILEGGK	509,28	53.46
	INILDQAVCR	601,33	59.70
	VFDYTEWIQS	644,31	33.50
	GGDECNINEHR	650,78	63.57
	ANINILDQAVCR	693,87	56.06
	FFCLSSK	887,42	27.98
	LQFGLH	714,4	0.36
	VLCAGILEGGK	1116,62	0.53
	EIYPGLLEK	1061,6	0.27
	GGDECNINEHR	1300,55	0.47
	INILDQAVCR	1201,65	0.58
	EIYPDVPHCANINILDQAVCR	2497,25	106
	IMGWGTISPTK	1190,58	97
	EWDKDIMLIR	1318,69	89
	VGGDECNINEHR	1399,63	66
L-amino acid oxidase 109254996	TDLPSR	344,69	42.52
	FLEIAK	360,72	37.34
	VQVHFN	372,2	35.36
	VVEELR	372,72	46.12
	NNPGLLK	378,23	36.46
	LEECFR	427,2	30.02
	FQALDFK	434,74	29.84
	STTDLPSR	438,73	52.49
	LYEESLR	455,24	35.23
	KEDWYAN	463,21	34.79
	DDRNPLEECFR	484,22	39.28
	VQVHFNAR	485,77	63.68
	NGLTATSNPK	501,77	69.47
	NGLTATSNPK	502,26	55.16
	VTVLEASER	502,28	63.94
	FFQALDFK	508,27	47.74
	IEIQQNDR	508,76	29.59

L-amino acid oxidase 109254996	QLYEESLR	519,27	38.59
	KFEPPLPPK	526,82	40.49
	NPLEECFR	532,75	63.67
	VIEIQQNDR	557,8	55.56
	HDDIFGYEK	562,26	65.71
	QVTVLEASER	566,31	55.87
	DLSLIHQLPK	582,35	53.82
	IKFEPPLPPK	583,36	58.93
	YILDKYDTY	597,3	48.07
	FHGWIDSTIK	602,32	46.29
	KHDDIFGYEK	626,31	40.49
	SAGQLYEESLR	626,82	72.26
	FWEDEGIHGGK	637,8	66.74
	DRNPLEECFR	668,32	48.77
	TDYEEFLEIAK	679,34	59.47
	EDIQTFCHPSK	681,32	60.62
	LKHDDIFGYEK	682,85	49.01
	TADYVIVCTTSR	693,35	60.77
	KFWEDEGIHGGK	701,85	66.34
	IYFAGEYTAQFH	723,85	56.56
	ETDYEEFLEIAK	734,86	41.69
	ETDYEEFLEIAK	743,86	76.00
	YILDKYDTYSTK	755,39	65.97
	FQENDNAWYFIK	787,88	55.36
	DCADIVINDLSLIHQ	863,44	52.79
	LNEDSGYYVSFIESLK	932,47	63.36
	NNPGLLK	755,45	0.31
	KEDWYAN	925,41	0.3
	KEDWYANLGP	1192,58	0.47
	SAGQLYEESLR	1252,63	0.53
	EDIQTFCHPSK	1361,63	0.5
	NEFFQENDNAWYFIK	1965,89	0.49
	LNEFFQENDNAWYFIK	2079,98	0.61
	ELNEFFQENDNAWYFIK	2208,05	0.59
	GLELNEFFQENDNAWYFIK	2378,16	0.54
	YPVKPSETGK	1105,6	64
	FHGWIDSTIK	1203,62	74
	KEDWYANLGPMR	1479,79	68
	GIGDDANFFQALDFK	1657,77	117
	KEDWYANLGPMRLPTK	1918,99	88
	DCADIVINDLSLIHQLPK	2007,05	109
	DCADIVINDLSLIHQLPK	2007,05	112
	FDEIVGGMDQLPTSMYEAIK	2244,11	115
	FGLELNEFFQENDNAWYFIK	2524,19	79
	EATVTYQTSANEMSSVTADYVIVCTTSR	3084,43	124
	EGNLSPGAVDMIGDLLNEDSGYYVSFIESLK	3332,65	214

phospholipase A2 109254990	AAAICFR	404,71	61.57
	IICGGDDQCK	583,26	51.91
	EIICGGDDQCK	647,77	54.51
	NLIQFETLILK	666,41	60.95
	GEIICGGDDQCK	676,29	59.99
	GNLIQFETLILK	694,91	62.60
	GNLIQFETLILK	695,41	38.91
	SEENGEIICGGDDQCK	905,88	70.31
	TYSEENGEIICGGDDQCK	1037,94	68.57
	GNLIQFET	921,48	0.29
	NLIQFETLILK	1331,81	0.55
	SAYGCYCGWGGHGR	1588,64	0.57
	RLPTENCQEEPEPC	1758,76	0.45
	LDTYTYSEENGEIICGGDDQCK	2569,05	0.56
	EIICGGDDQCK	1294,57	83
	LPTENCQEEPEPC	1602,67	101
	SAYGCYCGWGGHGRPQDATDR	2256,94	71
	LDTYTYSEENGEIICGGDDQCKK	2695,2	143
cysteine rich secretory protein 109254988	CIESHSPR	329,16	50.02
	QIVWYK	418,74	37.92
	IWHGENK	442,23	42.88
	WTEIIHI	456,26	39.87
	VDLHNSLR	477,27	54.11
	IVDLHNSLR	533,81	63.05
	IVDLHNSLR	534,3	47.41
	WTEIIHIW	549,3	56.63
	YTQIVWYK	550,8	64.40
	WTEIIHIWHGENK	554,96	54.06
	SATCFCQNK	558,24	41.31
	SVDFDSESPR	569,76	69.10
	GSVDFDSESPR	598,27	48.97
	VCQYCPAGNIR	669,32	49.93
	YGIGADPPNAVTGH	684,84	56.94
	EWYSEAAANAER	698,82	66.05
	AGCAAAYCPSLEY	716,81	40.08
	YVCQYCPAGNIR	750,85	58.89
	YGIGADPPNAVTGHY	766,37	69.67
	YGIGADPPNAVTGHYT	816,9	62.47
	CIESHSPR prolucid	985,46	0.42
	YGIGADPPNAVTGHYTQ	1761,85	0.53
	PPNAVTGHYTQIVWYK	1874,99	0.55
	SGPPCGDCPSACDNGLCTNP	2137,79	0.55
	AGCAAAYCPSLEYSYFYVC	2252,95	0.53
	SGPPCGDCPSACDNGLCTNPC	2297,84	0.59
	AAYCPSLEYSYFYVCQYCPAGNIR	2954,33	0.57

cysteine rich secretory protein 109254988	AAAYCPSLEYSYFYVCQYCPAGNIR	3024,36	0.58
	MEWYSEAAANAER	1527,62	117
	WTEIIHIWHGENK	1662,84	106
	QMQSDCSATCFCQNK	1847,62	118
	PPNAVTGHYTQIVWYK	1873,93	146
	SVDFDSESPRKPEIQNK	1975,94	90
	YGIGADPPNAVTGHYTQIVWYK	2450,18	98
	AGCAAAYCPSLEYSYFYVCQYCPAGNIR	3311,48	173
C-type lectin proteoform 1 109254974	LVSITYPILK	573,87	28.38
	SSYEGHCYR	579,74	27.82
	LEWTDGTK	949,47	0.43
	LVSITYPILK	1146,73	0.4
	SSYEGHCYR	1158,47	0.32
	EWSEEPECIVSK	1492,68	0.39
	ADLVWIGLR	1042,62	74
	TTDNQWIS	1120,57	68
	TTDNQWISRPC	1377,62	70
	EWSEEPECIVSK	1492,66	82
	DCPSDWSSYEGHCYR	1861,69	102
	HSSEEVDFLVSITYPILK	2077,1	114
	VFQQEMTWEAAEQFCAQQR	2387,09	58
C-type lectin proteoform 2 109254976	SHLVSFR	423,24	42.59
	HLVSFR	758,44	0.32
	SSEEVDFLVSLLK	1465,76	91
	HCPSGWYTYEGHCYR	1972,79	74
C-type lectin proteoform 3 109254978	GISYIWIGLR	589.85	52,26
	DCPSGWSSYEGH	1381,53	0.44
	DCPSGWSSYEGHCYK	1832,72	0.57
	GISYIWIGLR	1177,65	44
	SSYEGHCYKPFNEPK	1842,87	53
	WVNIYCGEINPFVCK	1898,94	67
	DCPSGWSSYEGHCYKPFNEPK	2545,09	129
vascular endothelial growth factor 109254982	SCVTVLR	834,45	0.24
	PSCVTVLR	931,5	0.41
	FTEHTECECR	1368,55	0.46
	QAMPFMEVFER	1367,6	74
	EMLVSILDEHPDEVAH	1833,78	139
	IEVMQFTEHTECECRPR	2222,01	79
	EMLVSILDEHPDEVAHLFR	2250,12	122
bradykinin potentiating protein 109254992	TPPAGPDVGPR	532,29	74.47
	TPPAGPDVGPR	1063,56	0.40
	DTPPAGPDVGPR	1178,59	0.28
	DHHSAPGGGGGGGGGGAR	1460,6	109
